# Thinking beyond blood: brain concentrations and brain–blood ratios in postmortem toxicology

**DOI:** 10.1093/jat/bkag083

**Published:** 2026-09-22

**Authors:** Kathrine Østerballe Skov, Mikayla Zoë van der Meer, Michael Nedahl, Marie Katrine Klose Nielsen

**Affiliations:** Section of Forensic Chemistry, Department of Forensic Medicine, Faculty of Health and Medical Sciences, University of Copenhagen, Copenhagen, Denmark; Section of Forensic Chemistry, Department of Forensic Medicine, Faculty of Health and Medical Sciences, University of Copenhagen, Copenhagen, Denmark; Section of Forensic Chemistry, Department of Forensic Medicine, Faculty of Health and Medical Sciences, University of Copenhagen, Copenhagen, Denmark; Section of Forensic Chemistry, Department of Forensic Medicine, Faculty of Health and Medical Sciences, University of Copenhagen, Copenhagen, Denmark

## Abstract

Toxicological analysis is an integral part of forensic death investigations. However, analysis is sometimes complicated by postmortem changes, and blood might not always be useable or available due to postmortem redistribution or degradation. In these cases, brain tissue can be useful as the brain is more protected against trauma, putrefaction, postmortem redistribution, skeletonization, and severe blood loss. However, reference concentrations of drugs in brain tissue are still scarce. This study aimed to report brain concentrations and brain–blood ratios for 122 compounds including drugs of abuse, pharmaceuticals, and metabolites frequently detected in forensic autopsy cases. In total, 2264 cases were collected over an 11-year period and retrospectively categorized as having either toxic or therapeutic levels of detected compounds based on blood concentrations. Toxic and therapeutic concentrations showed considerable overlap in both blood and brain due to drug tolerance and multi-drug combinations. Brain–blood ratios were highest for compounds with direct function on the central nervous system, especially antidepressants and sedative-hypnotics. The reported brain concentrations and brain–blood ratios can serve as reference values for interpretation of drug concentrations in brain tissue and the evaluation of cause of death in future forensic investigations.

## Introduction

The field of forensic toxicology is under continuous development through advances in analytical methods and improvements in interpretation of results to ensure quality and reliability of forensic results in the medico-legal investigation [1]. Postmortem toxicology is used in forensic investigations to assess potential drug use prior to death, therapeutic medicinal use, or the potential contribution of drugs to the cause of death [2]. However, postmortem drug concentrations are affected by several antemortem and postmortem effects, such as individual metabolism and excretion, postmortem redistribution, and decomposition [3–5]. These postmortem changes can complicate forensic investigations, and, in some cases, toxicological analysis must rely on other postmortem biological specimens because peripheral blood might not be available due to putrefaction, burns, or large blood losses [1, 2, 6–8].

Brain tissue is a useful alternative matrix for toxicological analysis in postmortem cases where peripheral blood is no longer available or viable for analysis [9–11]. Due to its more secluded position, the brain is more often protected against postmortem changes such as postmortem redistribution and decomposition. Additionally, many drugs of abuse and pharmaceuticals exert their effects through the central nervous system (CNS) leaving the brain as their site of action [12]. Following drug consumption and absorption, drug distribution from blood into specific tissue such as brain is determined by the affinity between drug and tissue components and drug transporters [13]. The blood–brain barrier (BBB) functions as one of the major protective barrier layers of the CNS and has an important regulative influence on drug delivery from the blood to the brain parenchyma [13, 14]. The BBB consists of endothelial cells forming capillary walls that extend throughout the brain parenchyma as a protective barrier against harmful substances such as neurotoxins and plasma proteins that can be damaging to neural tissue [14]. In addition, its barrier function consists of drug transporters such as the P-glycoprotein (P-gp) transporter and metabolic barriers expressing several drug-metabolizing enzymes acting as determining factors in drug access across the BBB [14, 15]. As such, brain penetration of a drug is dependent on several factors such as physiochemical properties and interaction with the P-gp transporter or drug-metabolizing enzymes such as cytochromes P450 at the BBB [16, 17]. Compounds intended to act within the CNS are typically designed to ensure high permeability across the BBB [15], which is facilitated through either lipid-mediated free diffusion or carrier- or receptor-mediated transport [18, 19]. Consequently, brain tissue is a relevant alternative matrix for toxicological analysis. However, interpretation of drug concentrations in brain tissue requires reference data where concentrations in brain and peripheral blood are compared [6]. Careful interpretation of toxicological results is fundamental in forensic investigations and reference concentrations observed under toxic and therapeutic conditions are important for interpretation of postmortem drug concentrations [4]. Comprehensive lists of reference drug concentrations in postmortem blood and median drug concentrations in postmortem matrices have previously been published [1, 20–25]. Additionally, reference brain concentrations and brain–blood ratios focusing on selected drugs of abuse, antipsychotics, and antidepressants have previously been published [26–36], but reference brain concentrations for many drugs of abuse and pharmaceuticals are still limited compared to reference concentration in blood. Moreover, most published case studies and compilations focus on high drug concentrations such as in lethal cases, but postmortem reference concentrations are also needed for therapeutic concentrations [1]. Thus, the aim of this article was to evaluate toxic and therapeutic brain concentrations and brain–blood ratios from frequently detected drugs, pharmaceuticals, and metabolites in forensic postmortem autopsy cases from Eastern Denmark.

## Materials and methods

The Department of Forensic Medicine, University of Copenhagen, carries out autopsies of decedents as part of forensic death investigations by local police authorities. The present study comprises retrospective data from 2264 autopsy cases subjected to toxicological investigations between January 2014 and March 2025. The dataset includes paired blood and brain concentrations of 122 compounds frequently detected in autopsy cases during toxicological analysis performed at the Section of Forensic Chemistry.

### Case selection and classification

Cases were included if quantified results were available for paired blood and brain samples, and if there were three or more cases per compound. Data for some published drugs of abuse, pharmaceuticals, and metabolites were also included if the number of cases exceeded the number of cases originally published [26–34]. The antipsychotics chlorprothixene, clozapine, olanzapine, and quetiapine were not included, as reference concentrations for these drugs were recently published [37].

All cases were retrospectively classified into two categories depending on whether the observed postmortem drug blood concentration was within toxic or therapeutic levels based on toxicological details and the final forensic medical statement, as ruled by the forensic pathologist. Cases were categorized as toxic if it was estimated that the blood concentration of the drug of abuse or pharmaceutical was within toxic ranges and/or had contributed to death either alone or in combination with other drugs of abuse, pharmaceuticals, or disease. Cases were categorized as therapeutic if the postmortem analyte blood concentration was within therapeutic ranges [20] and not considered to have any contribution to cause of death. Metabolites were categorized according to their parent drug, and the sum of the parent drug and active metabolite was used in the toxicological report to assess if a toxic concentration was reached. If a compound appeared in less than three cases in either the therapeutic or toxic category, the results for therapeutic and toxic were combined in one group.

### Collection of postmortem samples

Decedents were stored at 5°C and autopsy was typically performed within 4 days. During autopsy, biological specimens, including peripheral blood and brain tissue, were collected for toxicological investigation and stored at –20°C prior to analysis. Two femoral blood samples of 4–8 mL were collected without ligation of the iliac vein and stored in vacutainers with sodium fluoride and potassium oxalate. Brain tissue samples of 5–20 g were collected from the frontal cerebral cortex and stored with no preservatives.

### Sample preparation and toxicological analysis

Gray matter was manually separated from the brain tissue samples with a scalpel, and 500 mg was diluted (1:3) with LC–MS-grade water and homogenized using a gentleMACS dissociator (Milteney Biotic, Bergish Gladbach, Germany). The brain homogenate was centrifuged at 2000 *g* and 20°C for 10 minutes and transferred to new tubes. Whole blood and brain homogenate samples were prepared in duplicates using a fully automated sample preparation method on a Tecan Freedom EVO 200 robotic platform (Tecan, Männedorf, Switzerland) or a Hamilton Microlab Vantage 2.0 liquid handling system (Hamilton, Bonaduz, Switzerland) [38]. Following sample preparation, blood and brain homogenate samples were analyzed using a previously described comprehensive systematic toxicological analysis strategy comprising five multi-target methods with quantification by ultra-high performance liquid chromatography–tandem mass spectrometry (UHPLC–MS/MS) [39].

### Data processing

Results from quantitative analysis of drugs in postmortem blood and brain were extracted from the laboratory information management system (LIMS). Drugs of abuse, pharmaceuticals, and metabolites that had been quantified in at least three cases in both blood and brain were included and the data was presented as 10th–90th percentiles intervals and median values for the blood concentration, brain concentration, and brain–blood ratio.

## Results and discussion

A total of 2264 autopsy cases were included in the current study, providing paired concentrations and brain–blood ratios for 122 commonly encountered compounds in forensic cases. Those compounds consisted of 9 analgesic/anesthetics, 18 antidepressants, 11 antihistamines, 11 antipsychotics, 8 cardiovascular drugs, 16 opioids, 21 sedative-hypnotics, which included benzodiazepines, 9 stimulants, and 19 drugs that did not fit in one of the above categories. Methadone, paracetamol, and N-demethyldiazepam were the most frequently detected compounds. Intoxication was considered as the cause of death or to have contributed to death in 45% of cases with the majority of autopsy cases involving multiple drugs in both the toxic and therapeutic categories.

Of the 122 detected compounds, 59 compounds had sufficient data to allow classification into therapeutic and toxic categories, findings of 33 compounds were considered therapeutic, the findings of flubromazelam were classified as toxic, while classification was not possible for the remaining 29 compounds due to limited case numbers in one or both categories. Table 1 provides an overview of findings of all compounds distributed into nine drug groups, including their respective median values and 10th–90th percentiles in blood concentrations, brain concentrations, and brain–blood ratio. As shown in Table 1, a substantial overlap between the therapeutic and toxic categories was observed for both concentrations and brain–blood ratios. The median brain–blood ratio ranged from 0.07 to 26 across all compounds, where the lowest median brain–blood ratios (<0.01) were observed for ropivacaine and fexofenadine, and the highest median brain–blood ratios (>10) were observed for dextromethorphan, vortioxetine, scopolamine, norfluoxetine, and fluoxetine.

**Table 1:** Median and 10th–90th percentiles for blood and brain concentrations and brain–blood ratios for 122 compounds distributed into nine drug groups. For each case, the drug findings were categorized as toxic or therapeutic. Findings were combined in one group if there were less than three cases in either the toxic or therapeutic category.

Group	Drug	*N*	Category	Blood concentration (mg/kg)		Brain concentration (mg/kg)		Brain–blood ratio	
				Median	10th–90th percentile	Median	10th–90th percentile	Median	10th–90th percentile
Analgesic/anesthetic	Bupivacaine	11	Combined	0.84	0.38–3.2	0.68	0.25–1.7	0.68	0.42–0.90
	Gabapentin	8	Toxic	44	25–70	18	3.4–36	0.48	0.087–0.68
		50	Therapeutic	11	1.8–28	3.7	0.69–11	0.46	0.13–0.69
	Ibuprofen	11	Combined	13	9.0–90	6.0	5.0–17	0.47	0.35–0.90
	Ketamine	7	Toxic	1.1	0.21–1.8	0.82	0.3–4.1	1.3	0.83–2.3
		37	Therapeutic	0.33	0.084–0.86	0.40	0.075–3.1	1.5	0.58–6.7
	Lidocaine	199	Therapeutic	0.19	0.030–1.2	0.16	0.030–1.2	0.91	0.33–2.5
	Paracetamol	17	Toxic	290	130–1150	160	49–670	0.62	0.41–0.69
		414	Therapeutic	15	7.0–42	9.0	5.0–23	0.67	0.37–0.86
	Pregabalin	7	Toxic	17	5.5–45	5.2	2.1–9.6	0.29	0.17–0.60
		51	Therapeutic	5.0	1.5–11	1.7	0.60–3.6	0.35	0.17–0.53
	Ropivacaine	≤5	Therapeutic	3.5	1.1–29	0.26	0.20–0.34	0.070	0.022–0.36
	Salicylic acid	27	Combined	43	15–170	11	5.0–68	0.29	0.19–0.39
Antidepressant	Amitriptyline^a^	13	Toxic	1.2	0.30–5.3	7.1	1.0–37	4.1	2.6–12
		38	Therapeutic	0.14	0.031–0.49	0.44	0.11–1.5	3.4	2.4–5.8
	Citalopram^a^	48	Toxic	0.99	0.46–2.4	3.3	1.2–6.1	3.3	2.0–4.8
		114	Therapeutic	0.22	0.024–0.57	0.76	0.11–2.3	3.9	2.7–5.7
	N-Demethylcitalopram	44	Toxic	0.19	0.083–0.43	0.32	0.089–0.57	1.6	0.94–2.2
		58	Therapeutic	0.080	0.019–0.20	0.16	0.047–0.38	2.0	1.3–3.4
	Clomipramine	≤5	Therapeutic	0.25	0.23–0.42	1.0	0.62–2.7	4.0	2.6–6.1
	N-Demethylclomipramine	≤5	Therapeutic	0.38	0.13–0.49	2.8	0.97–3.4	7.4	7.0–8.1
	Duloxetine^a^	15	Toxic	0.25	0.087–0.35	1.8	0.69–3.4	7.9	5.2–12
		42	Therapeutic	0.081	0.024–0.24	0.69	0.20–2.1	8.3	5.7–14
	Fluoxetine	≤5	Toxic	2.1	0.55–6.2	19	4.6–22	5.6	4.0–11
		10	Therapeutic	0.31	0.043–0.81	3.3	0.46–9.3	12	8.7–20
	Norfluoxetine	≤5	Toxic	0.85	0.72–2.4	7.8	5.7–14	7.0	4.6–12
		10	Therapeutic	0.39	0.028–0.79	5.2	0.49–8.1	14	10–18
	Imipramine	9	Combined	0.18	0.073–0.81	0.49	0.33–3.6	4.0	2.6–6.8
	N-Demethylimipramine	9	Combined	0.078	0.029–0.76	0.48	0.23–4.7	6.3	5.7–14
	Mianserin	28	Combined	0.083	0.023–0.14	0.23	0.067–0.51	2.4	1.7–6.8
	Mirtazapine^a^	15	Toxic	0.76	0.17–2.4	1.2	0.24–6.6	1.8	1.2–4.6
		137	Therapeutic	0.12	0.031–0.34	0.19	0.047–0.59	1.6	1.0–2.9
	Nortriptyline	14	Toxic	0.53	0.34–1.87	4.3	2.4–15	6.8	3.7–13
		55	Therapeutic	0.12	0.027–0.76	0.96	0.22–6.2	8.9	5.8–14
	Paroxetine	10	Combined	0.23	0.003–2.3	3.4	0.041–25	12	7.7-18
	Sertraline^a^	36	Toxic	0.55	0.30–2.5	4.7	2.8–15	7.9	5.2–13
		125	Therapeutic	0.16	0.040–0.60	1.7	0.37–4.8	9.5	6.0–16
	Venlafaxine	27	Toxic	2.0	0.82–22	3.2	1.1–28	1.7	0.82–2.4
		52	Therapeutic	0.34	0.060–0.99	0.56	0.091–2.0	2.0	0.99–3.0
	O-Desmethylvenlafaxine	26	Toxic	0.88	0.21–5.7	1.1	0.22–4.0	1.3	0.77–1.7
		55	Therapeutic	0.36	0.088–1.2	0.54	0.16–1.6	1.7	1.2–2.3
	Vortioxetine	≤5	Combined	0.11	0.059–0.27	1.2	0.90–2.7	14	8.6–19
Antihistamine	Cetirizine	≤5	Toxic	0.55	0.21–0.69	0.15	0.065–0.25	0.35	0.29–0.37
		51	Therapeutic	0.15	0.072–0.54	0.049	0.028–0.18	0.38	0.28–0.61
	Chlorpheniramine	≤5	Therapeutic	0.013	0.003–0.023	0.063	0.014–0.11	5.0	3.7–5.7
	Cyclizine	≤5	Combined	0.24	0.055–14	0.26	0.093–50	2.3	1.1–4.5
	Desloratadine	7	Therapeutic	0.031	0.013–0.15	0.024	0.014–0.098	0.77	0.59–1.3
	Diphenhydramine	20	Therapeutic	0.017	0.002–0.26	0.045	0.007–0.48	3.2	1.9–5.1
	Doxylamine	≤5	Therapeutic	0.039	0.020–0.049	0.095	0.041–0.099	2.0	1.8–2.4
	Fexofenadine	≤5	Therapeutic	1.1	0.55–1.4	0.050	0.034–0.22	0.080	0.056–0.16
	Hydroxyzine	≤5	Toxic	0.27	0.21–0.54	1.0	0.62–3.5	3.9	2.9–6.1
		27	Therapeutic	0.035	0.003–0.24	0.18	0.014–0.92	4.9	3.3–9.0
	Loratadine	≤5	Therapeutic	0.003	0.002–0.005	0.004	0.003–0.010	2.1	1.6–2.2
	Meclizine	≤5	Therapeutic	0.008	0.008–0.02	0.06	0.027–0.21	3.8	2.4–26
	Promethazine	9	Toxic	0.23	0.058–0.94	0.95	0.26–4.5	4.1	2.7–7.4
		69	Therapeutic	0.079	0.019–0.34	0.36	0.082–1.5	4.7	2.6–7.1
Antipsychotic	Amisulpride	≤5	Combined	0.51	0.40–3.2	0.059	0.034–0.43	0.11	0.072–0.14
	Aripiprazole	≤5	Toxic	0.16	0.13–0.17	0.79	0.58–0.91	4.6	3.6–6.9
		38	Therapeutic	0.11	0.020–0.33	0.38	0.11–1.0	4.2	2.9–7.0
	Flupentixol	11	Combined	0.013	0.002–0.027	0.12	0.013–0.16	7.5	5.1–9.1
	Haloperidol	7	Toxic	0.040	0.020–0.055	0.083	0.054–0.11	2.9	1.1–5.0
		35	Therapeutic	0.009	0.003–0.029	0.049	0.023–0.11	5.9	2.9–11
	Levomepromazine	7	Combined	0.035	0.010–0.13	0.091	0.054–0.80	4.7	3.1–7.2
	Perphenazine	7	Combined	0.013	0.005–0.18	0.11	0.044–1.1	7.3	5.6–10
	Risperidone	20	Combined	0.005	0.003–0.018	0.013	0.007–0.030	2.4	0.96–4.1
	9-Hydroxyrisperidone	62	Combined	0.008	0.003–0.027	0.024	0.012–0.059	3.2	1.0–6.1
	Sertindole	≤5	Combined	0.19	0.042–0.68	0.98	0.23–3.3	5.3	4.9–8.0
	Ziprasidone	≤5	Combined	0.016	0.003–0.73	0.064	0.040–0.10	7.5	0.84–16
	Zuclopenthixol	≤5	Toxic	0.052	0.028–0.21	0.47	0.20–2.3	9.2	6.5–11
		34	Therapeutic	0.038	0.017–0.069	0.22	0.084–0.49	6.2	3.2–10
Cardiovascular	Amlodipine	6	Toxic	0.13	0.13–1.1	0.18	0.14–1.2	1.1	0.96–1.4
		100	Therapeutic	0.061	0.033–0.11	0.066	0.044–0.13	1.3	0.75–2.0
	Atenolol	≤5	Therapeutic	0.50	0.28–2.7	0.20	0.068–0.73	0.26	0.18–0.38
	Carvedilol	21	Therapeutic	0.026	0.010–0.11	0.030	0.007–0.099	0.96	0.42–2.0
	Enalaprilat	≤5	Combined	0.20	0.039-0.51	0.067	0.007-0.20	0.22	0.18-0.62
	Metoprolol	9	Toxic	1.9	0.75–56	1.7	1.1–48	0.89	0.67–1.9
		180	Therapeutic	0.12	0.021–0.46	0.16	0.036–0.76	1.8	0.70–3.0
	Propranolol	24	Combined	0.043	0.007–0.42	0.11	0.035–1.5	5.4	2.9–8.0
	Verapamil	≤5	Toxic	2.6	1.8–4.6	2.3	1.0–15	0.89	0.53–2.9
		15	Therapeutic	0.16	0.037–0.32	0.093	0.021–0.40	0.47	0.30–1.6
	Norverapamil	≤5	Toxic	1.2	0.42–1.8	0.47	0.21–2.8	0.63	0.44–1.5
		14	Therapeutic	0.13	0.047–0.21	0.053	0.030–0.11	0.49	0.33–0.88
Opioid	Buprenorphine	18	Toxic	0.002	0.0004–0.004	0.007	0.004–0.016	6.6	2.4–12
		20	Therapeutic	0.0008	0.0001–0.008	0.005	0.001–0.023	7.3	2.3–20
	Norbuprenorphine	7	Toxic	0.005	0.001–0.010	0.003	0.001–0.005	0.65	0.41–0.71
		≤5	Therapeutic	0.013	0.007–0.014	0.005	0.0003–0.006	0.48	0.27–0.57
	Codeine	16	Toxic	0.48	0.083–2.5	0.65	0.11–2.8	1.3	0.72–2.1
		85	Therapeutic	0.036	0.015–0.19	0.052	0.029–0.30	1.8	0.73–2.8
	Dextromethorphan	7	Combined	0.019	0.004-0.065	0.085	0.041-0.46	11	4.0-11
	Fentanyl	20	Toxic	0.012	0.002–0.086	0.022	0.003–0.14	1.7	0.96–5.1
		95	Therapeutic	0.004	0.001–0.023	0.011	0.003–0.064	2.8	0.85–11
	Ketobemidone	9	Toxic	0.11	0.024–0.29	0.16	0.032–0.41	1.3	1.1–1.5
		10	Therapeutic	0.041	0.013–0.077	0.038	0.022–0.15	1.6	0.84–2.1
	Loperamide	29	Therapeutic	0.005	0.0008–0.013	0.003	0.0008–0.008	0.74	0.44–1.1
	Methadone	363	Toxic	0.57	0.20–1.9	1.3	0.42–3.7	2.1	1.3–3.5
		94	Therapeutic	0.19	0.045–1.1	0.40	0.097–2.3	2.0	1.3–3.9
	6-MAM	22	Combined	0.017	0.006–0.098	0.090	0.033–0.33	5.5	1.2–12
	Morphine^a^	190	Toxic	0.19	0.042–1.0	0.13	0.035–0.61	0.78	0.23–1.8
		117	Therapeutic	0.072	0.017–0.35	0.059	0.027–0.24	0.91	0.37–2.9
	Morphine-6-glucuronide	11	Toxic	0.32	0.14–0.86	0.11	0.031–0.20	0.23	0.16–0.97
		10	Therapeutic	0.46	0.16–1.1	0.10	0.014–0.45	0.22	0.068–0.59
	Naloxone	12	Therapeutic	0.008	0.004–0.025	0.016	0.008–0.031	2.2	0.48–3.8
	Oxycodone	69	Toxic	0.33	0.037–2.1	0.56	0.063–2.5	1.8	0.99–2.5
		112	Therapeutic	0.048	0.009–0.24	0.086	0.018–0.42	1.8	1.2–2.9
	Tapentadol	≤5	Therapeutic	0.066	0.015–0.31	0.077	0.020–1.6	2.4	1.4–4.9
	Tramadol	65	Toxic	2.4	0.47–13	3.4	0.64–25	1.6	0.73–2.3
		106	Therapeutic	0.39	0.045–1.5	0.63	0.071–2.1	1.7	1.2–2.6
	O-Desmethyltramadol	67	Toxic	0.38	0.051–1.7	0.52	0.071–2.4	1.5	1.0–1.9
		75	Therapeutic	0.099	0.037–0.31	0.16	0.061–0.41	1.6	1.0–2.3
Sedative–hypnotic	Alprazolam^a^	55	Toxic	0.028	0.009–0.14	0.077	0.021–0.38	2.5	1.9–3.2
		55	Therapeutic	0.016	0.003–0.041	0.039	0.010–0.096	2.6	1.8–3.2
	Bromazepam^a^	29	Toxic	0.22	0.045–0.44	0.31	0.063–0.73	1.4	1.2–1.7
		35	Therapeutic	0.045	0.015–0.29	0.076	0.021–0.40	1.5	1.2–2.1
	Chlordiazepoxide^a^	53	Toxic	0.52	0.059–2.3	0.70	0.12–3.6	1.6	1.0–2.8
		96	Therapeutic	0.25	0.022–1.5	0.40	0.032–2.2	1.5	0.87–2.5
	Clobazam	≤5	Therapeutic	0.017	0.010–0.041	0.041	0.021–0.082	2.0	1.9–2.3
	Norclobazam	≤5	Therapeutic	0.066	0.036–0.13	0.16	0.11–0.28	2.5	2.2–3.3
	Clonazepam^a^	64	Toxic	0.005	0.002–0.022	0.033	0.013–0.099	6.6	2.6–13
		43	Therapeutic	0.004	0.002–0.011	0.028	0.014–0.076	7.0	3.7–14
	7-Aminoclonazepam^a^	167	Toxic	0.15	0.027–0.48	0.11	0.023–0.32	0.74	0.54–1.1
		171	Therapeutic	0.063	0.018–0.18	0.045	0.016–0.14	0.76	0.57–1.1
	Demoxepam	59	Toxic	0.83	0.21–3.1	1.8	0.27–4.6	1.6	1.2–2.2
		96	Therapeutic	0.57	0.23–2.2	0.91	0.36–3.9	1.5	1.1–2.1
	Diazepam^a^	59	Toxic	0.082	0.020–0.35	0.13	0.028–0.40	1.4	0.91–2.2
		148	Therapeutic	0.041	0.010–0.18	0.064	0.015–0.32	1.5	0.86–3.1
	N-Demethyldiazepam^a^	110	Toxic	0.14	0.020–0.48	0.31	0.047–1.1	2.3	1.3–3.6
		310	Therapeutic	0.051	0.009–0.31	0.13	0.021–0.68	2.4	1.3–3.9
	Flubromazolam	≤5	Toxic	0.008	0.005–0.012	0.043	0.043–0.049	6.4	4.0–9.0
	Lorazepam	17	Combined	0.017	0.004-0.096	0.058	0.017-0.30	3.5	2.3-5.6
	Midazolam	65	Therapeutic	0.015	0.005–0.31	0.099	0.017–0.91	3.8	1.4–13
	Nitrazepam^a^	≤5	Toxic	0.019	0.009–0.098	0.18	0.10–0.37	9.6	5.1–12
		≤5	Therapeutic	0.008	0.006–0.012	0.045	0.017–0.063	5.3	2.0–7.3
	7-Aminonitrazepam^a^	23	Toxic	0.073	0.035–0.41	0.062	0.023–0.32	0.83	0.58–0.95
		36	Therapeutic	0.062	0.018–0.15	0.043	0.015–0.14	0.81	0.53–1.2
	Oxazepam^a^	45	Toxic	0.027	0.011–0.39	0.064	0.022–0.75	2.0	1.4–3.3
		126	Therapeutic	0.047	0.013–0.37	0.11	0.028–0.73	2.0	1.2–3.3
	Phenobarbital	≤5	Toxic	14	7.8–66	17	9.8–91	1.3	1.2–1.5
		12	Therapeutic	11	6.1–22	12	7.2–29	1.2	1.1–1.4
	Scopolamine	≤5	Therapeutic	0.0003	0.0003–0.0006	0.009	0.003–0.011	26	5.8–39
	Temazepam^a^	17	Toxic	0.022	0.011–0.065	0.026	0.012–0.067	1.1	0.81–2.2
		24	Therapeutic	0.026	0.013–0.050	0.025	0.015–0.063	0.96	0.59–1.9
	Zolpidem	7	Toxic	0.29	0.13–0.96	0.28	0.098–1.8	0.96	0.78–1.8
		11	Therapeutic	0.11	0.066–0.57	0.086	0.048–0.33	0.73	0.49–1.2
	Zopiclone	62	Toxic	0.18	0.030–0.83	0.28	0.031–1.2	1.4	0.80–2.1
		129	Therapeutic	0.031	0.008–0.13	0.045	0.012–0.20	1.5	0.99–2.4
Stimulant	Amphetamine^a^	50	Toxic	0.45	0.080–2.4	1.3	0.18–6.2	2.8	0.98–4.0
		83	Therapeutic	0.10	0.024–0.93	0.32	0.065–2.2	3.2	1.7–4.6
	Atomoxetine	8	Combined	0.098	0.037-0.36	0.73	0.21-2.4	5.9	5.0-9.3
	Cocaine^a^	76	Toxic	2.6	0.10–13	7.5	0.11–25	2.4	0.70–5.0
		134	Therapeutic	0.084	0.015–0.52	0.18	0.027–0.84	2.0	0.73–4.9
	Benzoylecgonine	34	Toxic	4.1	0.56–11	1.8	0.48–4.9	0.49	0.30–1.0
		178	Therapeutic	0.32	0.072–1.7	0.20	0.056–0.79	0.66	0.35–1.3
	Caffeine	18	Therapeutic	4.6	1.1–15	5.1	1.0–16	1.1	0.98–1.3
	MDA	10	Toxic	0.064	0.017–0.13	0.19	0.043–0.45	2.9	1.9–4.5
		9	Therapeutic	0.032	0.019–0.11	0.15	0.068–0.33	3.2	2.4–5.0
	MDMA^a^	14	Toxic	1.2	0.19–4.6	4.2	0.55–23	2.6	2.0–4.8
		13	Therapeutic	0.26	0.059–0.57	0.77	0.26–3.6	3.7	1.7–5.5
	Methamphetamine	6	Combined	0.50	0.12–4.1	1.0	0.50–8.6	3.1	1.5–4.1
	Methylphenidate^a^	≤5	Toxic	0.020	0.0085–0.44	0.062	0.017–0.71	2.0	1.6–3.9
		44	Therapeutic	0.027	0.0073–0.21	0.058	0.015–0.37	2.6	0.91–4.0
Miscellaneous	Atropine	30	Therapeutic	0.013	0.005–0.056	0.013	0.006–0.029	0.91	0.38–3.0
	Baclofen	15	Combined	0.68	0.20–3.3	0.11	0.032–0.58	0.18	0.084–0.32
	Biperiden	12	Therapeutic	0.010	0.001–0.034	0.012	0.005–0.038	1.4	0.62–5.2
	Colchicine	≤5	Combined	0.010	0.006–0.037	0.003	0.002–0.007	0.28	0.20–0.36
	Donepezil	6	Therapeutic	0.14	0.086–0.17	0.30	0.19–0.33	2.0	1.8–2.8
	Ephedrine^a^	59	Therapeutic	0.070	0.018–0.38	0.16	0.049–0.80	2.6	1.6–4.1
	Fluconazole	≤5	Therapeutic	2.3	0.34–15	1.9	0.28–11	0.82	0.73–1.0
	Lamotrigine	22	Therapeutic	4.2	0.55–11	6.5	1.1–11	1.4	1.1–1.9
	Levetiracetam	13	Therapeutic	9.6	1.4–19	11	1.7–26	1.2	0.91–1.7
	LSD	≤5	Combined	0.001	0.0003–0.002	0.011	0.003–0.011	8.7	5.8–18
	Metformin	22	Combined	3.6	1.2–28	1.0	0.31–5.6	0.28	0.062–0.56
	Metoclopramide	13	Toxic	0.26	0.20–0.79	0.20	0.095–0.50	0.82	0.33–1.3
		49	Therapeutic	0.12	0.015–0.35	0.071	0.013–0.21	0.76	0.34–1.3
	Ondansetron	30	Therapeutic	0.030	0.014–0.065	0.054	0.021–0.14	2.1	1.0–3.1
	Orphenadrine	≤5	Toxic	2.6	2.0–5.4	11	6.4–35	4.1	3.2–6.3
		21	Therapeutic	0.39	0.059–1.5	1.1	0.15–3.9	3.0	2.3–4.6
	N-Demethylorphenadrine	14	Combined	0.098	0.028–0.53	0.63	0.13–2.2	5.1	3.2–7.3
	Sildenafil	7	Therapeutic	0.12	0.026–0.23	0.050	0.005–0.097	0.41	0.18–0.82
	THC	245	Therapeutic	0.005	0.001–0.018	0.007	0.002–0.033	1.81	0.54–4.8
	THC-COOH	248	Therapeutic	0.016	0.005–0.041	0.012	0.005–0.041	0.95	0.43–1.9
	Topiramate	≤5	Therapeutic	8.8	2.8–13	6.3	1.7–10	0.63	0.45–0.89

a Drugs previously published with fewer cases by the Department of Forensic Medicine [26–34].

### Brain–blood ratio

The brain–blood ratio of a given drug or metabolite is affected by several factors, including lipophilicity, pKa, stability, half-life, and postmortem redistribution. Additionally, route of administration and time from consumption to death are important factors influencing the brain–blood ratio [19]. The distribution of compounds and their corresponding median brain–blood ratios within each drug group without classification into toxic or therapeutic categories is shown in Fig. 1.

**Figure 1:**
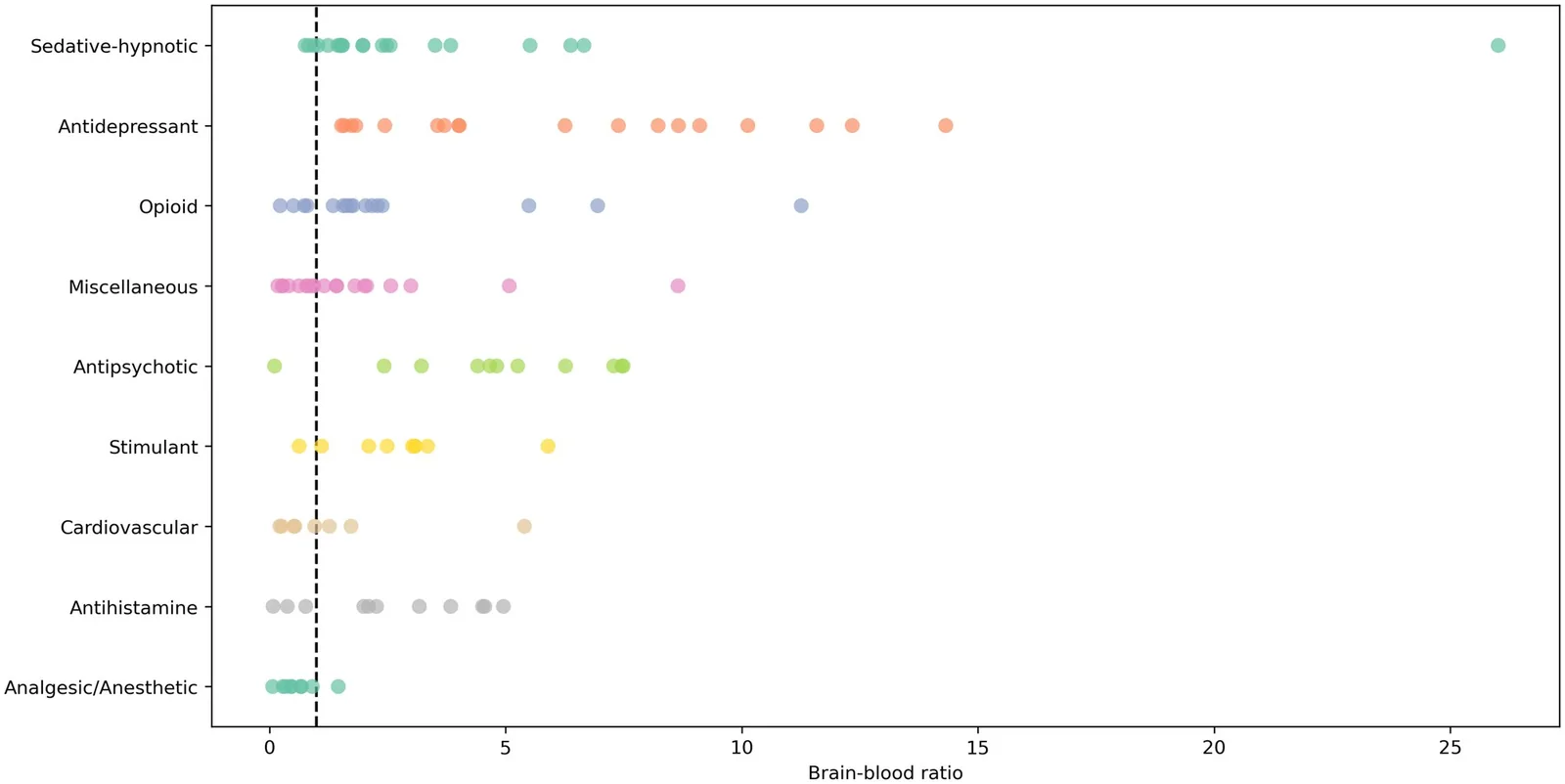
Distribution of all 122 compounds, including drugs of abuse, pharmaceuticals, and metabolites, and their corresponding median brain–blood ratio grouped into 9 representative drug groups. Median brain–blood ratios are based on all cases and not divided into toxic and therapeutic categories. The dashed line (**- - -)** designates the distribution of brain–blood ratios above and below 1.

As seen in Fig. 1, the highest and widest distributions of median brain–blood ratios were observed for compounds primarily targeting the nervous system, especially antidepressants and sedative-hypnotics (Fig. 1). Antidepressants, antipsychotics, and stimulants all had a median brain–blood ratio above 1 except for amisulpride and the metabolite benzoylecgonine, indicating their permeability across the BBB. The detected antidepressants showed a wide range of brain–blood ratios with vortioxetine having the highest brain–blood ratio of 14 within this group. The sedative-hypnotics showed the widest range from 0.76 to 26 of median brain–blood ratios due to the high brain–blood ratio of scopolamine. The remaining sedative-hypnotics were more clustered in the lower end of the range from 0.76 to 6.7. In the analgesic/anesthetic group, ketamine was the only drug with a higher concentration in brain than blood.

Generally, the observed variation in brain–blood ratios across compounds was consistent with differences in their permeability across the BBB, influenced by lipophilicity, polarity, and volume of distribution, as well as variation in Pgp-mediated efflux at the BBB or nonspecific receptor binding of certain compounds [16, 40–46].

### Metabolites

Drug metabolites may be inactive or possess similar, enhanced, or different pharmacological actions as their parent drug [47]. Differences in brain–blood ratios for parent drugs and their corresponding metabolites were observed across drugs represented in Table 1. For several drugs, the brain–blood ratios were higher than those observed for their metabolites. This was seen for, e.g. cocaine and its metabolite benzoylecgonine as well as nitrazepam and clonazepam and their corresponding 7-amino metabolites. Despite the higher ratios for the parent drugs, both benzoylecgonine and the 7-amino metabolites exhibited higher median concentration in both blood and brain than their respective parent drugs. The higher concentration of 7-amino metabolites compared to parent compound has previously been reported in blood, pericardial fluid, muscle, and vitreous humor [1, 48]. A similar difference in brain–blood ratio was observed for buprenorphine, citalopram, hydroxyzine, and their metabolites, where the median brain–blood ratios of the parent drug were up to 10 times higher than the median brain–blood ratio of active metabolite. In contrast, clomipramine, imipramine, and fluoxetine exhibited lower median brain–blood ratios than their metabolites N-demethylclomipramine, N-demethylimipramine, and norfluoxetine. However, these findings were based on a limited number of cases in each category.

Differences in brain–blood ratios could be explained by differences in physicochemical properties of the compounds, BBB permeability, metabolism and elimination kinetics, and postmortem changes [49, 50]. Higher concentrations of the parent drug relative to its metabolite might indicate a recent exposure and limited metabolic conversion, whereas higher concentrations of the metabolite may be consistent with a longer interval between drug intake and death [50]. Nevertheless, the parent drug-metabolite ratio is not a specific indicator of the time since drug intake, as it may also be influenced by metabolite accumulation during repeated exposure, differences in elimination rates, and postmortem conversion or degradation of the parent drug through bacterial or enzymatic activity [49, 50]. Therefore, the parent drug-metabolite ratio should be interpreted with caution in the context of drug pharmacokinetics and case-specific information.

### Interpretive considerations in postmortem toxicology

The interpretation of postmortem drug concentrations and their contribution to the cause of death is a complex task that depends on multiple physiological, environmental, pharmacological, and postmortem factors [51–53]. Therefore, a drug concentration measured after death may not necessarily reflect the concentration at time of death [5, 52–57].

One major limitation is that drug concentrations can continue to change after death due to ongoing postmortem processes. As the postmortem interval increases, biochemical and structural changes in tissues may affect drug stability and distribution within the body [2, 58]. Some compounds are prone to postmortem degradation, including chemical, enzymatic, or bacterial degradation [59]. For unstable compounds, this process can lead to a reduction in detectable parent drug concentrations or in some cases, formation of metabolites or other degradation products that complicate interpretation. This is particularly relevant for compounds such as cocaine, olanzapine, and nitrobenzodiazepines [49, 55, 60]. Simultaneously, postmortem redistribution may occur, whereby concentration gradients between tissues and blood may lead to passive diffusion of compounds, often resulting in elevated concentrations in central blood compared to peripheral sites, thereby affecting the brain–blood ratio [5, 57, 61]. Compounds with high lipophilicity, large volumes of distribution, or basic properties are particularly prone to postmortem redistribution [2, 62]. Therefore, the anatomical site of sampling must be carefully considered when interpreting toxicological results [59, 62].

Even when postmortem changes are limited, the relationship between drug concentration and toxicity is rarely straightforward. Concentrations considered toxic in drug-naïve individuals may be within the therapeutic range in habitual users due to tolerance development associated with chronic drug use, as seen for opioids and stimulants [4, 63]. For example, habitual users and individuals using methadone for maintenance therapy or with a history of addiction may develop a higher tolerance than naïve recreational users [64]. Consequently, lower doses can be fatal in cases of non-habitual use causing overlapping postmortem methadone concentrations between fatal cases and habitual users, especially when combined with other drugs [64]. Combined drug effects may be additive or synergistic, particularly when CNS depressants are involved [52, 65]. These interactions may result in fatal outcomes even when individual drug concentrations fall within therapeutic or sub-toxic ranges. Additionally, underlying diseases or pathological conditions such as cardiovascular disease, respiratory disorders, or hepatic impairment may increase susceptibility to drug toxicity or contribute directly to death. In such cases, the cause of death may involve a complex interplay between toxicological and natural factors [52]. Therefore, interpretation of postmortem drug concentrations requires particular caution for compounds associated with tolerance development or additive effects. Measured concentrations should be interpreted in conjunction with other toxicological findings, as well as information from the autopsy report and relevant circumstantial data [50].

In summary, postmortem drug concentrations and consequently the brain–blood ratios, are affected by multiple factors, including the timing of drug intake relative to death, the postmortem interval, postmortem degradation, and the extent of postmortem redistribution. These factors must be considered alongside interindividual variability in drug tolerance and the potential additive or synergistic effects of co-occurring compounds. By providing new reference concentrations and brain–blood ratios, this study strengthens the framework for future toxicological interpretation of the cause of death.

## Conclusion

The present study reports toxic and therapeutic postmortem brain concentrations and brain–blood ratios for 122 drugs of abuse, pharmaceuticals, and metabolites frequently encountered in forensic autopsy cases. For the majority of the compounds, brain concentration exceeded those in blood, consistent with expectations for compounds that act on the CNS. The reported brain concentrations and brain–blood ratios may serve as reference values for the interpretation of toxicological findings in future forensic death investigations, when brain tissue is used as a supplementary matrix or when blood is unavailable for analysis.

## Data Availability

The data underlying this article cannot be shared publicly due to the nature of the study, which involves retrospective analysis of postmortem material collected from routine cases. Data sharing is limited to protect confidentiality and is per institutional guidelines.
